# Stress Tolerance of *Methylobacterium* Biofilms in Bathrooms

**DOI:** 10.1264/jsme2.ME12146

**Published:** 2012-12-01

**Authors:** Takehisa Yano, Hiromi Kubota, Junya Hanai, Jun Hitomi, Hajime Tokuda

**Affiliations:** 1 R&D—Safety Science Research, Kao Corporation, Akabane, Ichikai, Haga, Tochigi 321–3497, Japan; 2 R&D—Household Products, Kao Corporation, Minato, Wakayama-shi, Wakayama, 640–8580, Japan

**Keywords:** bathroom, pink biofilm, *Methylobacterium*, tolerance to cleaning agent components, tolerance to drying

## Abstract

A comprehensive survey of microbial flora within pink biofilms in bathrooms was performed. Pink biofilms develop relatively rapidly in bathrooms, can be difficult to remove, and are quick to recur. Bacterium-sized cells were found to be predominant in 42 pink biofilms in Japan using a scanning electron microscope. *Methylobacterium* strains were detected from all samples in bathrooms by an isolation method. To explain this predominance, 14 biofilm samples were analyzed by fluorescence *in situ* hybridization. *Methylobacterium* was indicated to be the major genus in all biofilms. The isolated *Methylobacterium* survived after contact with 1.0% cleaning agents, including benzalkonium chloride for 24 h. Their tolerance did not differ under biofilm-like conditions on fiber reinforced plastics (FRP), a general material of bath tubs, floors, and walls. Also, the strains exhibited higher tolerance to desiccation than other isolated species on FRP. Some *Methylobacterium* survived and exhibited potential to grow after four weeks of desiccation without any nutrients. These specific characteristics could be a cause of their predominance in bathrooms, an environment with rapid flowing water, drying, low nutrients, and occasional exposure to cleaning agents.

In bathrooms and kitchens, microorganisms face survival difficulties, such as rapid flowing water, drying, low nutrients, and occasional exposure to cleaning agents; however, microorganisms thrive (11, 37, 53), often as biofilms (8, 31), microbial communities that attach to biotic or abiotic surfaces (8, 49, 50). Biofilms in moist environments often exhibit pink (10, 13, 23) or black (15, 20, 24) pigmentation.

Notably, pink biofilms in bathrooms (Fig. 1) recur more rapidly than other forms of staining and are difficult to remove under dry conditions. These characteristics have led to pink biofilms being the second most common microbial stain in bathrooms in Japan after fungal black biofilms, according to our survey from 2006 to 2007.

Several studies have focused on the microbial composition of pink biofilms in bathrooms, such as on shower curtains (23) or shower heads (10). Some microorganisms with pink-red pigmented colonies have been isolated, including pink-pigmented yeasts, the genus *Rhodotorula*, and bacteria, the genera *Methylobacterium*, *Brevundimonas*, and *Rhodobacter*, suggesting that these species affect the colors of the biofilms (13). Notably, the genus *Methylobacterium*, a pink pigmented facultative methylotroph (16), has been also isolated from tap water (13) and human feet (3) and mouths (2); however, the predominant species and the reason why biofilms in bathrooms are pink remain unclear. Studying them would help to clarify the mechanisms by which the microorganisms adapt to these severe conditions; therefore, we focused on pink biofilms in bathrooms and investigated the microbial flora. Using an electron microscope and fluorescence *in situ* hybridization (FISH), we studied whether one or multiple genera are dominant in biofilms. We then searched for the factors responsible for their predominance by examining tolerance to cleaning agents and desiccation stress.

## Materials and Methods

### Strains and culture conditions

The strains used were bacteria isolated from pink biofilms in bathrooms: *Methylobacterium mesophilicum* KMC10, *Methylobacterium radiotolerans* KMC5, *Methylobacterium fujisawaense* KMC4, *Brevundimonas vesicularis* KMC13, *Candidatus Chryseobacterium massiliae* KMC14, *Rhodococcus corynebacteroides* KMC15, *Chryseobacterium gregarium* KMC16, *Rhodococcus* sp. KMC17, *Rhodococcus* sp. KMC18, *Roseomonas mucosa* KMC19, *Burkholderia cepacia* KMC20, *Deinococcus grandis* KMC21, *Microbacterium arborescens* KMC22. *Roseomonas mucosa* KMC19 and *Microbacterium arborescens* KMC22 were cultivated with R2A broth (Becton Dickinson, Sparks, MD), *Burkholderia cepacia* KMC20 was cultivated with Soybean Casein Digest broth (Becton Dickinson), and the other bacteria were cultivated with Potato Dextrose broth (Becton Dickinson) for 3 d at 30°C.

### Scanning electron microscopy (SEM)

The biofilms were sampled from 42 points in the bathrooms of 14 houses in Japan with toothpicks, and fixed for 3 d at room temperature by adding 5 mL glutaraldehyde, followed by dehydration by successive 50, 70, 90, 99, and 100% (v/v) ethanol washes (3 min each), dried, sputtered with platinum/palladium (Pt-Pd) with a sputter coater (E-1030 ion spatter; Hitachi, Tokyo, Japan), and stored at room temperature. The specimens were then examined with a scanning electron microscope (S4300SE/N; Hitachi) operated at 7 to 15 kV.

### Fluorescence *in situ* hybridization (FISH) assay

The FISH assay was performed as described elsewhere (1, 7, 33) with some modifications. The biofilms on the surfaces of shampoo bottles and shower baskets for storing soap and sponges were removed by slicing with a box cutter, and fixed for 2 h at 4°C by adding 5 mL of 3% (v/v) paraformaldehyde. Subsequently, the fixatives on the sliced abiotic surfaces were picked up and washed gently (so as not to disturb the biofilm structure) with phosphate-buffered saline (PBS). The biofilms were then placed in plastic cases and embedded by gently introducing 20% (w/v) acrylamide. The acrylamide was allowed to polymerize at 30°C for 1 h. The embedded biofilms were carefully lifted from the cases and cut into 1-inch sections.

For hybridization, a EUB338 probe, specific to the domain bacteria (1), and MB probe, specific to the genus *Methylobacterium* (40), were used. The probes are listed in Table 1. Oligonucleotide probes labeled with fluorescein isothiocyanate (FITC) or tetrame-thylrhodamine 5-isothiocyanate (TRITC) were purchased from Hokkaido System Science (Hokkaido, Japan). Competitor probes to ensure specificity (30) and helper probes to enhance signal intensity (12) were generally used together with the fluorescent probe in cases of low specificity. Because MB alone gave low fluorescence, a competitor probe and helper probe were constructed (Table 1) and used to obtain high fluorescence. Simultaneous hybridization with probes that required different stringency conditions was performed; hybridization with the probe requiring higher stringency was performed first, followed by that with the probe requiring lower stringency. All samples were simultaneously stained with calcofluor white (32) for 10 min in the dark to determine β(1–3) and β(1–4)-linked glucosyl polymer-containing exopolysaccharides and fungal distribution. For microscopy and image analyses, a model LSM510 META confocal laser scanning microscope (CLSM; Carl Zeiss, Oberkochen, Germany) equipped with a diode laser (405 nm), Ar ion laser (458 and 488 nm) and HeNe ion laser (543 nm) was used. All images were combined and processed with Imaris 5 software. The biomasses of three representative biofilms were quantified using Comstat2 (17, 48) under the Image J shell, and the proportion of MB was calculated as follows; (MB biomass)/(MB biomass + EUB338 biomass + calcofluor white biomass) × 100. The average proportion was determined by using 10 representative microscopic images of each sample.

### Isolation of pink microorganisms

The samples were collected from 42 pink biofilms in Tokyo, Wakayama, and Tochigi, Japan. To isolate microorganisms, the pink biofilms in bathrooms were sampled with toothpicks, streaked onto Potato Dextrose Agar (PDA; Becton Dickinson), Nutrient Agar (NA; Becton Dickinson), and R2A Agar (R2AA; Wako Pure Chemical, Osaka, Japan), and cultured at 30°C for bacterial colonies and 25°C for fungal colonies.

### Sequencing and phylogenetic analyses

Template DNA samples for use in PCR were prepared as follows: a single colony of the isolate on solid growth medium was removed with a sterile toothpick and placed in 1 mL MilliQ water. The cell suspension was heated to 100°C for 10 min, and the lysate was used in PCR. Approximately 500-bp 16S rRNA gene sequences were amplified with a Microseq 500 16S rRNA gene PCR module (PE Applied Biosystems). The reaction mixture (50 μL) contained 25 μL diluted genomic DNA and 25 μL ready reaction mixture. The reaction profile for the amplification was initial denaturation at 95°C for 10 min, 30 cycles of 95°C for 30 s, 60°C for 30 s, and 72°C for 45 s, a final extension at 72°C for 10 min, and a 4°C soak. The PCR products were purified with a High Pure PCR Product Purification kit (Roche Diagnostics, Mannheim, Germany) according to the manufacturer’s directions. The purified DNA was recovered in 25 μL deionized water. The amplified 16S rRNA gene was subjected to cycle sequencing with the Microseq module. The reaction mixture (20 μL) contained 3 μL purified PCR product, 4 μL deionized water, and 13 μL sequencing reaction mixture (forward and reverse sequencing mixture in separate reactions). The cycling conditions were 96°C for 10 s, 50°C for 5 s, and 60°C for 4 min, followed by a 4°C soak. The cycle-sequenced DNA was precipitated with a DyeEx 2.0 spin kit (Qiagen, Tokyo, Japan) according to the manufacturer’s instructions, and finally analyzed with an ABI Prism 3130 genetic analyzer (PE Applied Biosystems). The sequence data were compiled with DNASIS-Pro software (Hitachi Software Engineering, Tokyo, Japan). The fragments were subjected to homology-based searches of the APORON database (Techno Suruga Laboratory, Shizuoka, Japan) and phylogenetic trees were constructed to ascertain the phylogenetic positions of the isolates. In constructing phylogenetic trees, species related to identified species, which were found in previous reports (27, 42, 46), were added to the database.

### Pink biofilm formation assay

Although there are various biofilm formation assays (36), we imitated the bathroom environment to construct a novel biofilm model. The bacteria isolated from pink biofilms were grown under the indicated culture conditions, centrifuged for 5 min at 7,000×g, and washed and resuspended in water of 3.5° DH (CaCl_2_; 52.0 mg L^−1^, MgCl_2_6H_2_O; 31.8 mg L^−1^) to an OD_600_ of 0.5. Circular holes 1.2 cm in diameter were made on a 5 mm thick silicone sheet (As One Co., Osaka, Japan), and the sheet was attached to a fiber reinforced plastics (FRP) sheet (Engineering Test Service Co., Osaka, Japan), a major material for bath tubs, by pressure bonding. Some 500 μL prepared microorganisms were inoculated into the pores of the silicone sheet on the FRP. After 24 h of incubation at 30°C, water was completely removed and incubation continued for 24 h at 30°C. Finally, the silicone sheet was removed. To observe bacterial viability in models of pink biofilms on FRP, a *Bac*Light LIVE/DEAD bacterial viability staining kit (Molecular Probes, Eugene, OR) was used as previously described (51) with some modifications. A 100 μm-thick silicone sheet with circular holes 1.2 cm in diameter was attached to the model without covering the pigmented part. Two stock solutions of stain (SYTO 9 and propidium iodide) were each diluted to 3 μM with water of 3.5° DH, and 5 μL of the mixed solution was added to the samples. A cover glass was attached by instant adhesive, and observation made by CLSM.

### Cleaning agent susceptibility assay in test tubes

The following agents were used: alkyl dimethyl benzyl ammonium chloride (BAC [Sanisol C]; Kao Corporation, Tokyo, Japan), sodium dodecyl sulfate (SDS; Wako Pure Chemical), Triton X-100 (Sigma Chemical, St. Louis, MO), and butyl diethylene glycol (BDG; Tokyo Kasei Kogyo, Tokyo, Japan). All agents were prepared with water of 3.5° DH supplemented with 50 mM HEPES buffer, adjusted to pH 7.4, and diluted to 5.0, 1.0, and 0.1% (w/v). The bacteria were grown in PDB for 3 d at 30°C, centrifuged for 5 min at 7,000×*g*, washed in water of 3.5° DH, and resuspended in water of 3.5° DH to an OD_600_ of 0.8. The suspension were added to the assay mixtures at a ratio of 1 to 100 and incubated for 5 min, 120 min, and 24 h. After the reaction had been stopped by the addition of Diluent with Lecithin & Polysorbate 80 solution (LP; Wako Pure Chemical) at as much as ten times the volume of the reaction mixture, 3 μL was spotted onto PDA for KMC4, KMC5, KMC10, KMC13, KMC14, KMC15, KMC16, KMC17, KMC18, and KMC21, R2AA for KMC19 and KMC22, and SCDA for KMC20 and incubated for 3 d at 30°C to observe their colonies to clarify the minimal concentrations of agents at which the bacteria could not grow.

### Cleaning agents susceptibility assay on FRP

After pink biofilms had formed, 500 μL of 5.0% BAC (pH 7.4) was added and incubated for 5 min. After the agents had been removed, 500 μL water of 3.5° DH was added and removed. The biofilms on FRP sheets were then transferred into 50 mL centrifuge tubes with 10 mL LP and 5.0 g glass beads, 1.5–2.5 mm in diameter, and vortexed vigorously for 1 min to remove the bacteria from the sheets. LP containing bacteria was diluted, spread on each medium as indicated for the bactericidal assay in tubes, and incubated at 30°C for 3 d.

### Desiccation tolerance assay

Although there are various desiccation assays (19), we imitated the bathroom environment to construct a novel model. In the pink biofilm formation assay, the microorganisms on FRP were incubated for 10 d at 30°C after water had been removed. The surviving bacteria were quantified as described in the bactericidal activity assay of biofilms.

### Nucleotide sequence accession numbers

The 16S gene sequence data of the isolated *Methylobacterium* were deposited in the DNA Data Bank of Japan (DDBJ) under serial accession numbers AB629723 to AB629736.

## Results

### SEM observation

**Fig. 2:**
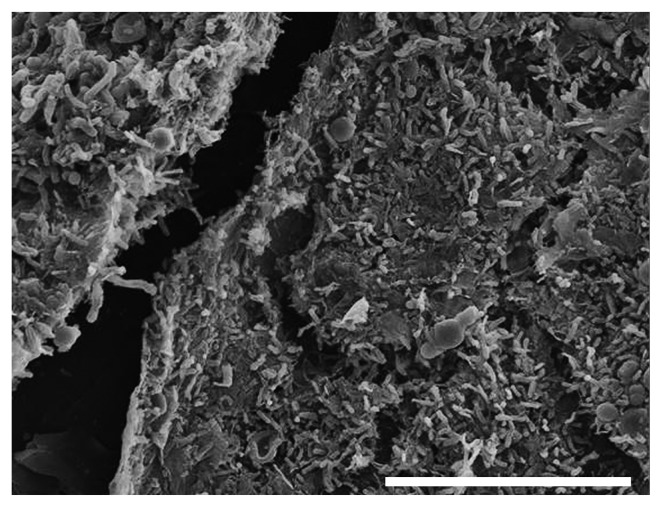
Representative pink biofilm observed by SEM. Bar, 20 μm.

For a detailed characterization by SEM, 42 pink biofilms were investigated. Some 24% of the biofilms were wet and slimy, but the others were dry. Nineteen percent were near drains, 31% were on walls, on floors, and around doors or windows, and 50% were on in-bath products including chairs, bottles, and brushes. Biofilms generally develop a mushroom or mat-like structure (17, 51). All the biofilms observed had a mat-like structure, and Fig. 2 shows a typical image. Interestingly, yeast-sized cells were rarely observed (12%) and were few in number when they were. Instead, rod-shaped microorganisms, 0.3 to 0.5 by 0.9 to 1.4 μm, were predominant in all the biofilms tested. These biofilms contained round microbial clumps (17%) and networks of fungal filaments (12%), as well as the clumps of rod-shaped microorganisms. In rather wet areas such as the bathroom drains, slimy networks, which would be extracellular polymeric substances, were also found (9.5%). In this investigation, the biofilms were directly fixed with glutaraldehyde without any incubation, because some microorganisms in biofilms could have proliferated, changing the population ratio before observation during the long complicated processes of sample preparation.

### Isolation of *Methylobacterium* from pink biofilms

We isolated 1,691 colonies from 42 pink biofilms. To identify microorganisms that could contribute to the pigmentation of the biofilms, approximately 500-bp sequences of 16S rRNA gene in colonies colored yellow, orange, and brown, as well as pink and red (405 colonies) were compared to sequences in GenBank. The colonies were identified as comprising numerous genera, including *Methylobacterium*, *Brevundimonas*, and *Roseomonas* (Table 2), and *Methylobacterium* strains were isolated from each biofilm. *Rhodotorula* were isolated from 17 of the 42 biofilms.

### FISH assay

**Fig. 3:**
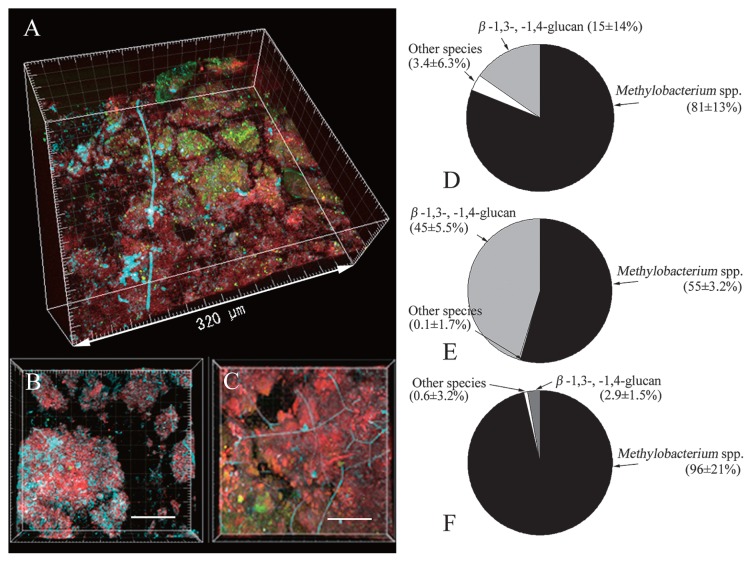
FISH assay and microbial community composition of pink biofilms. A–C, CLSM photomicrographs showing the spatial distribution of microorganisms in three independent pink biofilms. The organisms were targeted by *in situ* hybridization with the ROX-labeled probe MB and FITC-labeled probe EUB338, and simultaneously stained with calcofluor white. Cells of MB-stained *Methylobacterium* are red; cells of EUB338-stained bacteria are green. Cells containing β-1,3-glucan like fungi are aqua blue. D–F, pie charts of *Methylobacterium*, other bacteria, and β-1,3-, 1,4-glucans. *Methylobacterium* is the bacterial group that hybridized with MB, other bacteria are the bacterial group that hybridized with EUB338, and β(1–3) and β(1–4)-linked glucosyl polymers are the positions that hybridized with calcofluor white. Values are the mean ± standard deviation for duplicate samples.

We then analyzed the microbial communities using FISH to clarify if the dominant cells were *Methylobacterium*. The spatial distribution of the genus *Methylobacterium* and other bacteria was visualized and quantified by simultaneous *in situ* hybridization with fluorescence-labeled 16S rRNA gene targeting probes (Fig. 3). EUB338 was reported to be insufficient for the detection of some bacteria (9), but all the microorganisms in the pink biofilms were dyed with the two probes; therefore, these two probes were considered sufficient for staining pink biofilms. Calcofluor white, which specifically stains β(1–3) and β(1–4)-linked glucosyl polymers, major polysaccharides in cell walls of yeasts and other fungi, was also used simultaneously to identify yeast cells. Fig. 3A, B, C are typical images of the biofilms: Fig. 3A is a typical image of a biofilm in which bacteria other than *Methylobacterium* were relatively frequently observed; Fig. 3B is a typical image in which polysaccharide stained with calcofluor white was frequently observed; Fig. 3C shows that almost all surfaces were covered with *Methylobacterium*. We took ten photographs of a sample of each typical type, and their average biomasses were compared (Fig. 3D, E, F). All the results indicated that *Methylobacterium* was predominant in the biofilms; therefore, we concluded that *Methylobacterium* was predominant in pink biofilms.

### Phylogenetic analyses of *Methylobacterium* isolates

**Fig. 4:**
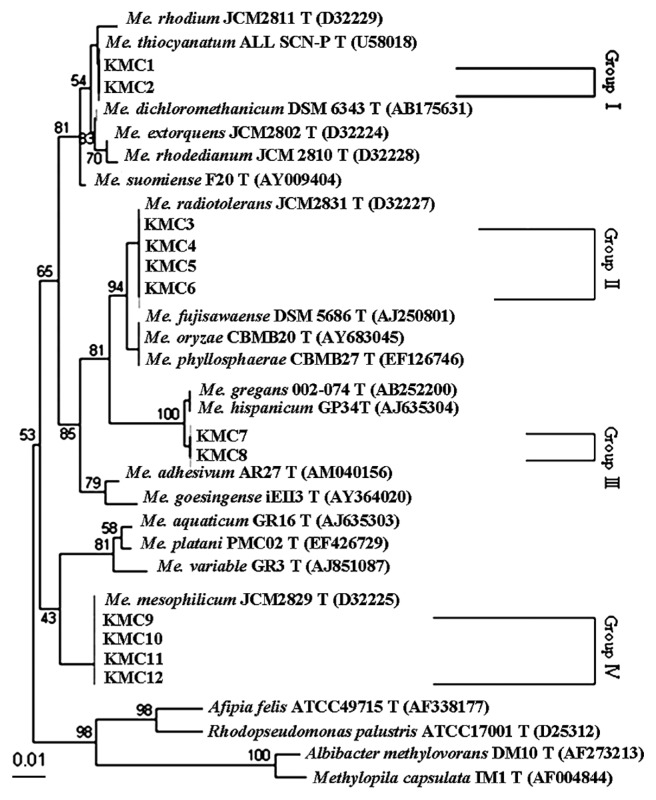
Phylogenetic tree based on approximately 500 bp of the 16S rRNA gene sequences of *Methylobacterium* isolates from bathrooms using the neighbor-joining method. The data for type strains of *Methylobacterium* and other genera were from GenBank. Bootstrap percentages (>50%) based on 100 replications are given at branch points. Bar, 0.01 changes per nucleotide position.

A phylogenetic tree of representative *Methylobacterium* strains was constructed (Fig. 4). The *Methylobacterium* isolates were divided into four groups, and *Methylobacterium mesophilicum* KMC10, *Methylobacterium fujisawaense* KMC4, and *Methylobacterium radiotolerans* KMC5 were used as typical *Methylobacterium* isolates in subsequent experiments..

### Pink biofilm formation assay

**Fig. 5:**
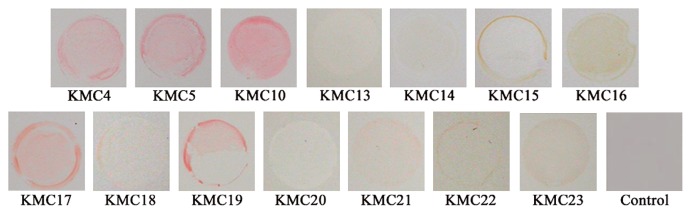
Model biofilms formed on FRP sheets. Photographs show the model biofilms with strain names below the photographs. KMC10, KMC4, and KMC5 indicate the biofilms of *Methylobacterium*. The control is FRP not inoculated with bacteria.

Model biofilms of various isolated strains including the *Methylobacterium* were formed to clarify whether they were pink. After being resuspended in water of 3.5° DH, the general degree of water hardness in Japan, the isolated bacteria were incubated on FRP. Typical biofilms 24 h after the water had been removed are shown in Fig. 5. The biofilms of the genus *Methylobacerium* were similar to those observed in bathrooms in color, but those of other species including *Rhodococcus* sp. KMC17 and *Roseomonas mucosa* KMC19 exhibited similar characteristics.

### Susceptibility to cleaning agents in test tubes

To investigate the tolerance to the components of cleaning agents, susceptibility to benzalkonium chloride (BAC), sodium dodecyl sulfate (SDS), polyoxyethylene p-*t*-octylphenyl ether, and diethylene glycol *n*-buthyl ether (BDG) was tested for the *Methylobacterium* and other microorganisms isolated from the biofilms (Table 3). The surviving numbers of isolated *Methylobacterium* were significantly higher than those of other bacteria.

### Susceptibility to cleaning agents on FRP

To investigate the susceptibility of the isolated bacteria under biofilm-like conditions, bactericidal activity assays were conducted against model biofilms by inoculating 10^7^–10^8^ cells onto each FRP sheet (Table 4), because biofilm bacteria are generally more tolerant of stress than planktonic bacteria (49). Some biofilms like those formed by *Rhodococcus* sp. KMC17 were removed only after water was added. Even in biofilms not washed away by water, the surviving numbers of the genus *Methylobacterium* were significantly higher than those of other bacteria under biofilm conditions. Vigorous vortexing with beads was used to remove bacteria from FRP sheets and the surviving number was investigated. The loss of surviving cells was less than one log, meaning that the treatment would not have significantly affected the results. The FRP sheets were also observed by a confocal laser scanning microscope (CLSM). Aggregates that attached to the sheets were not observed, meaning that the vortexing was sufficient to remove the microbes from FRP sheets.

### Desiccation tolerance of the Methylobacterium

**Fig. 6:**
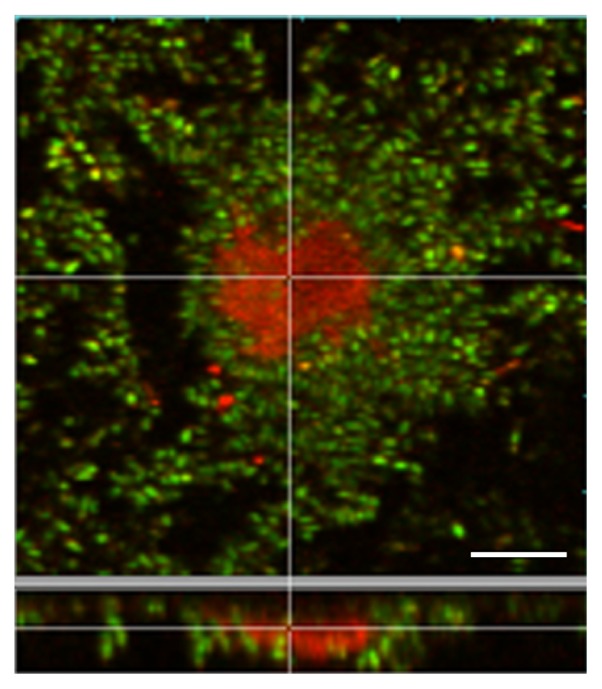
CLSM photographs showing viability within microcolonies in biofilms containing *Methylobacterium mesophilicum* KMC10. The cells were visualized using the LIVE/DEAD *Bac*Light kit. Green fluorescent cells are viable, whereas red fluorescent cells are dead. The results are representative of three experiments. Bar, 10 μm.

We examined the desiccation tolerance of the isolated strains on FRP by inoculating 10^7^–10^8^ cells onto each FRP sheet. Ten days after drying, the reduction in the survival of *Methylobacterium* was less than one log. The drying of other strains, however, led to values below the detection limit (Table 5). The result indicated that *Methylobacterium* is tolerant of drying in bathrooms. The biofilms were stained with a LIVE/DEAD *Bac*Light kit, and observed with CLSM (Fig. 6). Red cells were located inside the microcolony, and almost all other cells were alive.

## Discussion

In this study we examined pink biofilms in bathrooms by microscopic and quantitative analyses and clarified that the genus *Methylobacterium* predominated. To our knowledge, this is the first conclusive investigation of various pink biofilms in bathrooms and the first detailed investigation of why *Methylobacterium* predominated. Pink yeasts, including the genus *Rhodotorula*, have been also reported to be isolated from pink biofilms (15). Moreover, detailed microscopic analyses of various biofilms revealed the number of yeast-sized cells to be far smaller than the numbers of the genus *Methylobacterium*, indicating that *Rhodotorula* is not the predominant species in the biofilms.

*Methylobacterium* have been isolated from various environments, including soil (29), rivers (5), tap water (13), humans (2), and aquatic sediment (31), suggesting that it would not be unimaginable for the bacteria to be found in bathrooms; however, the finding that many other species exist simultaneously in bathrooms raised the question of why *Methylobacterium* was predominant.

Interestingly, yeasts, bacteria other than the *Methylobacterium*, and fungi were found on biofilms of the *Methylobacterium* without exception (Fig. 3). Previous reports that some *Methylobacterium* attach to plant roots (16, 22, 25, 26) and coaggregate with other species (43, 47) led us to speculate that *Methylobacterium* aggregated and attached to solid surfaces in the bathrooms, after which other microorganisms attached to them, and therefore survived even rapidly flowing water. Aggregation activities can be partially evaluated with a bioassay that tests the ratio of aggregated bacteria after incubation (47). In this assay, however, *Methylobacterium* did not necessarily exhibit more marked activities than other species (data not shown).

*Methylobacterium* might have other characteristics to survive in bathroom environments. One possibility is tolerance to cleaning agents. Tolerance of *Methylobacterium* to cleaning agents has not been reported, but some *Methylobacterium* were reported to tolerate high concentrations of chlorine (18). The results showed that *Methylobacterium* were more tolerant of the agents tested than other isolated strains. In addition, there was a similar tendency on FRP sheets. In a previous report of nosocomial outbreaks from inadequate antiseptics, bacteria were tolerant to at most 0.1–0.2% of BAC (52); therefore, *Methylobacterium* in the microbial flora was considered to be over 10 times more tolerant than previously reported strains, although the mechanism of tolerance remains unclear.

The bactericidal mechanism of BAC has been reported to be based on disruption of the membrane structure, followed by a proton imbalance and the accumulation of active oxygen species (14, 21, 34, 45). Regarding membrane permeability, the lipid composition of methane oxidizers, including the genus *Methylobacterium*, is unique in several respects. Methyl sterols, which are rarely observed in bacteria, have been shown to be present, and *Methylobacterium* possess a system of paired peripheral membranes (also called intracytoplasmic membranes) and are predominantly composed of monounsaturated C18 fatty acids (16, 38). Such systems may affect the delay in the permeation of BAC. The structure of the peripheral membrane was confirmed by observing the cytoplasm of *Methylobacterium mesophilicum* KMC10 with a transmission electron microscope (data not shown), although the relevance to permeability remains unclear and further study is needed. The quite high anti-oxidizing activities of carotenoids would also contribute to the tolerance to cleaning agents. Carotenoids could scavenge and prevent the formation of free radicals induced by BAC.

The third reason why *Methylobacterium* were predominant is their desiccation tolerance. There are repeated wet-dry cycles in bathrooms. Dry conditions are not normally suitable for the survival of microorganisms and it is considered that the longer the dry condition, the fewer microorganisms survive. Therefore, only highly desiccation-tolerant bacteria such as the genus *Methylobacterium* could survive in bathrooms; however, the reason why these bacteria are highly tolerant to drying remains unclear. Dry stress generally causes dysfunction of enzymes and/or the electron transport chains, and subsequent lipid peroxidation, protein denaturation, and mutation of DNA (4). The increased van der Waal’s interactions between phospholipids, followed by an increase in the phase transition temperature (T_m_) of membranes, could be an underlying mechanism (41). A higher T_m_ results in the aggregation of proteins, leakage and loss of solutes from cells (41). *Methylobacterium* could have certain superior evasion systems, such as polysaccharide secretion (6, 39), because it was reported that exopolysaccharide contributes to the desiccation tolerance of bacteria (35, 44); therefore, we searched the pink biofilms by staining them with calcofluor white to detect β(1–3) and β(1–4)-linked glucosyl polymers. Some biofilms were stained, but others were not (data not shown), indicating that a large amount of glucan does not play a crucial role in survival in bathrooms. Also, glucans were not detected in the *Methylobacterium* used in the desiccation tolerance experiments (data not shown). More studies are needed to clarify whether monosaccharides, proteins, or small amounts of polysaccharide contribute to desiccation tolerance, as well as antioxidants such as carotenoid. Concerning the localization of dead cells (Fig. 6), similar phenomena have been reported in various biofilms (28, 51) and were observed without any desiccation treatments; therefore, the biofilm development process might have led to cell death, and not the desiccation process.

There remain many other possible explanations for why *Methylobacterium* predominated. For example, it is possible that greater numbers of *Methylobacterium* than other bacteria or fungi invade bathrooms. This is because *Methylobacterium* have been reported to frequently occur in humans (2, 3) and human-made environments (10, 13, 23).

Our results suggest that the specific characteristics mentioned above could lead *Methylobacterium* to predominate. On the other hand, bathrooms also provide specific characteristic environments: rapid water flow, dry conditions, low nutrients, occasional exposure to cleaning agents, and so on. These characteristics could lead particular bacteria, *Methylobacterium*, to predominate. Further study is expected to clarify the role of *Methylobacterium* in the microbial ecology of bathrooms.

## Figures and Tables

**Fig. 1:**
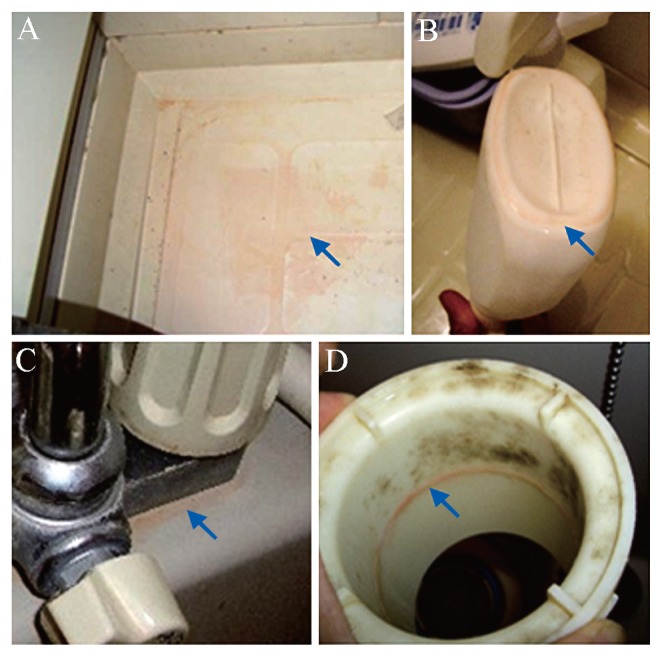
Pink biofilms in bathrooms. Arrows indicate biofilms on the floor (A), on the bottom of a bottle (B), around the connection between the faucet feed line and its housing (C), and inside the drain (D).

**Table 1:** Oligonucleotide probes

Probe	Sequence (5′ to 3′)	FA^a^ (%)	Specificity	Reference
EUB338	GCTGCCTCCCGTAGGAGT	20	Most Bacteria	(1)
MB	AGCGCCGTCGGGTAAGA	30	Genus *Methylobacterium*	(40)
Comp MB^b^	AGCGCCGTCTGGTAAGA	—	Competitor for MB	this study
Help MB^c^	CCAACTCCCATGGTGTGACGG	—	Helper for MB	this study

a FA, formamide concentration in the hybridization buffer.

b Unlabeled probe MB used as a competitor to enhance specificity.

c Unlabeled probe MB used as a helper to enhance specificity.

**Table 2:** Representative strains isolated from pink biofilms

Strain designation	Closest type strains	Accession No.^a^	% Sequence similarity^b^
*Methylobacterium* species			
KMC10	*Methylobacterium mesophilicum* JCM2829^T^	AB629729	99.3
KMC4	*Methylobacterium fujisawaense* DSM5686^T^	AB629731	99.1
KMC5	*Methylobacterium radiotolerans* JCM2831^T^	AB629725	99.3
Other species			
KMC13	*Brevundimonas vesicularis* IAM12105^T^	AB021414	99.8
KMC14	*Chryseobacterium taiwanense* BCRC17412^T^	EU336941	100
KMC15	*Rhodococcus corynebacteroides* DSM20151^T^	AD430066	99.6
KMC16	*Chryseobacterium gregarium* DSM19109^T^	AY230767	96.3
KMC17	*Rhodococcus* sp. DSM20151^T^	AF430066	99.4
KMC18	*Rhodococcus qingshengii* djl-6^T^	JF937542	99.8
KMC19	*Roseomonas mucosa* MDA5527^T^	AF538712	97.8
KMC20	*Burkholderia cepacia* ATCC25416^T^	AF097530	99.3
KMC21	*Deinococcus grandis* DSM3963^T^	Y11329	95.3
KMC22	*Microbacterium arborescens* DSM20754^T^	X77443	100
KMC23	*Brevundimonas nasdae* GTC1043^T^	AB071954	99.2

a Accession numbers based on 16S rRNA gene partial gene sequences.

b Sequence similarity (16S rRNA gene, approximately 500 bp) was searched using Aporon DB-FU 2.0. (Technosuruga Lab., Shizuoka, Japan)

**Table 3:** Susceptibility of isolated bacteria in test tubes to cleaning agent components

Strains	Minimal concentrations (%) of agents required for four log reduction for three exposure times^a^											

	Cleaning agents											

	BAC			SDS			BDG			Triton X-100		
			
	5 min	2 h	24 h	5 min	2 h	24 h	5 min	2 h	24 h	5 min	2 h	24 h
*Methylobacterium* strains												
KMC10	>5.0	>5.0	<0.10	>5.0	>5.0	>5.0	>5.0	>5.0	>5.0	>5.0	>5.0	>5.0
KMC4	>5.0	>5.0	1.0	>5.0	>5.0	>5.0	>5.0	>5.0	>5.0	>5.0	>5.0	>5.0
KMC5	0.10	1.0	0.10	>5.0	>5.0	>5.0	>5.0	>5.0	>5.0	>5.0	>5.0	>5.0
Other strains												
KMC15	<0.10	<0.10	<0.10	<0.10	<0.10	<0.10	>5.0	>5.0	>5.0	>5.0	>5.0	1.0
KMC21	<0.10	<0.10	<0.10	<0.10	<0.10	<0.10	>5.0	>5.0	1.0	<0.10	<0.10	<0.10
KMC14	<0.10	<0.10	<0.10	<0.10	<0.10	<0.10	>5.0	>5.0	>5.0	>5.0	>5.0	1.0
KMC16	<0.10	<0.10	<0.10	<0.10	<0.10	<0.10	>5.0	>5.0	>5.0	>5.0	>5.0	1.0
KMC20	<0.10	<0.10	<0.10	<0.10	<0.10	<0.10	>5.0	>5.0	1.0	>5.0	>5.0	>5.0
KMC18	1.0	<0.10	<0.10	>5.0	>5.0	1.0	>5.0	>5.0	>5.0	>5.0	>5.0	>5.0
KMC17	<0.10	<0.10	<0.10	<0.10	<0.10	<0.10	1.0	<0.10	<0.10	<0.10	<0.10	<0.10
KMC19	<0.10	<0.10	<0.10	>5.0	1.0	1.0	>5.0	>5.0	>5.0	>5.0	>5.0	>5.0
KMC22	<0.10	<0.10	<0.10	>5.0	1.0	1.0	>5.0	>5.0	>5.0	>5.0	>5.0	>5.0
KMC23	<0.10	<0.10	<0.10	<0.10	<0.10	<0.10	>5.0	>5.0	>5.0	>5.0	>5.0	>5.0
KMC13	<0.10	<0.10	<0.10	<0.10	<0.10	<0.10	<0.10	<0.10	<0.10	>5.0	>5.0	>5.0

a After 0.1, 1.0, and 5.0% agents had been mixed with each culture and incubated for 5 min, 2 h, and 24 h, surviving cells were detected by subsequent dilution, spotted on agar, and incubated.

**Table 4:** Susceptibility of isolated bacteria on FRP sheets to cleaning agent components

Strains	Log survival (log CFU/FRP sheet)	

	Water^a^	BAC^b^
*Methylobacterium* species		
KMC10	7.15	7.30
KMC4	7.52	7.27
KMC5	7.78	7.68
Other species		
KMC15	<2.00	<2.00
KMC21	6.58	<2.00
KMC20	2.90	<2.00
KMC14	<2.00	<2.00
KMC16	7.18	<2.00
KMC18	7.51	<2.00
KMC17	5.30	<2.00
KMC19	2.78	<2.00
KMC22	2.90	<2.00
KMC23	2.78	<2.00
KMC13	2.60	<2.00

a Log survival numbers of various biofilms formed on FRP after water had been applied for 5 min.

b Log survival numbers of various biofilms formed on FRP after 5.0% BAC had been applied for 5 min

**Table 5:** Desiccation tolerance

Strains	Log survivals (log CFU/FRP sheet)
*Methylobacterium* species	
KMC10	6.15
KMC4	6.26
KMC5	6.38
Other species	
KMC15	3.40
KMC14	<2.00
KMC16	2.78
KMC18	4.67
KMC17	<2.00
KMC21	<2.00
KMC20	<2.00
KMC19	<2.00
KMC22	3.88
KMC23	<2.00
KMC13	<2.00
